# Primary anastomosis and suturing combined with vacuum-assisted abdominal closure in patients with secondary peritonitis due to perforation of the small intestine: a retrospective study

**DOI:** 10.1186/s12893-023-02179-0

**Published:** 2023-09-15

**Authors:** Pooya Rajabaleyan, Rie Overgaard Jensen, Sören Möller, Niels Qvist, Mark Bremholm Ellebaek

**Affiliations:** 1 Research Unit for Surgery, Odense University Hospital, University of Southern Denmark, Odense, Denmark; 2 OPEN, Open Patient data Explorative Network, Department of Clinical Research, Odense University Hospital, University of Southern Denmark, Odense, Denmark

**Keywords:** Secondary peritonitis, Vacuum-assisted closure, Anastomotic leakage, Enteroatmospheric fistula, Open abdomen

## Abstract

**Background:**

Intestinal resection and a proximal stoma is the preferred surgical approach in patients with severe secondary peritonitis due to perforation of the small intestine. However, proximal stomas may result in significant nutritional problems and long-term parenteral nutrition. This study aimed to assess whether primary anastomosis or suturing of small intestine perforation is feasible and safe using the open abdomen principle with vacuum-assisted abdominal closure (VAC).

**Methods:**

Between January 2005 and June 2018, we performed a retrospective chart review of 20 patients (> 18 years) with diffuse faecal peritonitis caused by small intestinal perforation and treated with primary anastomosis/suturing and subsequent open abdomen with VAC.

**Results:**

The median age was 65 years (range: 23–90 years). Twelve patients were female (60%). Simple suturing of the small intestinal perforation was performed in three cases and intestinal resection with primary anastomosis in 17 cases. Four patients (20%) died within 90-days postoperatively. Leakage occurred in five cases (25%), and three patients developed an enteroatmospheric fistula (15%). Thirteen of 16 patients (83%) who survived were discharged without a stoma. The rest had a permanent stoma.

**Conclusions:**

Primary suturing or resection with anastomosis and open abdomen with VAC in small intestinal perforation with severe faecal peritonitis is associated with a high rate of leakage and enteroatmospheric fistula formation.

**Trial registration:**

The study was approved by the Danish Patient Safety Authority (case number 3-3013-1555/1) and the Danish Data Protection Agency (file number 18/28,404). No funding was received.

## Background

Faecal peritonitis due to perforation or anastomotic leakage of the small intestine is a severe condition, with a mortality rate ranging from 10 to 30% [1–5]. Intestinal resection to achieve source control and placement of a proximal stoma, with either closure of the distal intestinal segment or the placement of a distal stoma, is the conventional treatment of choice [5–10]. A primary anastomosis with a proximal protective diverting loop-ostomy may be an alternative option [11]. This strategy involving stoma placement carries a risk of high stoma output, short-bowel syndrome, permanent stoma, or complications to a later reversal [6].

Primary anastomosis/suturing with an open abdomen or damage control surgery with vacuum-assisted closure may be alternative options. Primary anastomosis and or associated bowel resection may be a considerably time-consuming procedure in some cases, and in this situation a physiological very unstable patient may benefit more from damage control surgery [12]. The establishment of an open abdomen with vacuum-assisted closure (VAC) was initially introduced in trauma settings to prevent and address the lethal triad of hypothermia, acidosis, and coagulopathy. The technique has increasingly been used to treat complicated secondary peritonitis [2, 13–25]. At the scheduled re-laparotomy, it is possible to perform lavage of the abdominal cavity, inspect the intestines and perform necessary additional surgical procedures to prevent serious complications. Enteroatmospheric fistula (EAF) formation and incomplete fascial closure are potential disadvantages [2, 13, 19, 26–32].

This study aimed to assess whether primary anastomosis or suturing was safe in patients with faecal peritonitis caused by a small intestinal perforation or anastomotic leakage using the open abdomen principle with VAC.

## Materials and methods

### Patient information

We performed a retrospective chart review of 20 patients (> 18 years) operated between January 2005 and June 2018 for faecal peritonitis caused by small bowel perforation and treated with primary intestinal resection and anastomosis or primary suturing and subsequent open abdomen with VAC. All patients had diffuse peritonitis in at least 3 of out of the 4 abdominal quadrants. Patients with perforation of the stomach, duodenum, and colon were excluded. Data retrieved from medical records included age, sex, body mass index (BMI), location and cause of intestinal perforation, duration of VAC treatment, number of VAC changes, the negative pressure applied, 90-day mortality rate and whether the patient had a stoma or not at discharge and 1-year follow-up. In addition, we recorded postoperative complications within 30 days, using the Clavien-Dindo classification index [33].

### Surgical approach

For the open abdomen treatment with VAC, the VAC® Abdominal Dressing System (KCI Vacuum Assisted Closure, San Antonio, TX, USA) was applied with a non-mesh mediated fascial traction technique [28]. After covering the intestinal loops with the visceral protective layer, the first layer of foam was placed into the wound and extended 5 cm subperitoneally from the wound edge. Another piece of foam was folded and placed in the laparostoma, allowing the foam to stick out above the abdominal wall, resembling a “shark fin”. We then loosely applied an occlusive drape in 10–15 cm wide strips. The fascial edges were simultaneously approximated manually towards the midline while applying negative pressure. The negative pressure applied was to the discretion of the operating surgeon. Dressings were changed within approximately 48 h, depending on clinical conditions. Fascial closure was commenced as early as possible depending on the clinical and paraclinical parameters, either in one or repeated sessions with interrupted nonabsorbable sutures (Prolene 2 − 0, Ethicon, New Jersey, United States) [27]. All operations were done by senior surgeons.

### Statistical analysis

Continuous data were presented as medians and ranges and compared using the Wilcoxon rank-sum test. Categorical data are presented as counts and proportions with binomial 95% confidence intervals (CI), using Stata software version 17.0 (StataCorp LP®, Texas, USA).

## Results

Twenty consecutive patients were included. The median age was 65 years (range: 23–90 years). Twelve patients were female (60%). The intestinal perforation was due to iatrogenic injury after previous gynaecological or gastrointestinal surgery in 16 cases, ileus in two cases, blunt trauma in one case, and an ischaemic perforation in one patient. Seventeen patients were treated with resection and primary anastomosis and three with suturing of the perforation (Table 1).

**Table 1:** Demographics, location and cause of intestinal perforation, type of repair, negative pressure with VAC in patients with and without anastomotic leakage

		Anastomotic leakage N = 5	No anastomotic leakage N = 15
**Gender**	Female	3	9
	Male	2	6
**Age years ** **median [range]**		64 [54–73]	66 [23–90]
**BMI**	BMI > 30	2	0
	BMI < 30	3	15
**Cause of peritonitis**	Secondary to abdominal or gynaecological surgery	5	11
	Ileus	0	2
	Ischaemia	0	1
	Trauma	0	1
**Vacuum, negative pressure (mmHg)** **median [range]**		75 [25–75]	50 [25–75]

VAC treatment was continued for a median of 4 days (range: 1–12 days). Following secondary closure of the abdomen, three patients (16%) required one or more additional laparotomies due to suspected or confirmed complications (Table 2).

**Table 2:** Postoperative surgical complications in patients according to the Clavien-Dindo classification [7]. Multiple complications may have occurred in the same patient

Clavien-Dindo classification	2	3a	3b	4a, 4b and 5
**Fascial dehiscence**	0	0	3	0
**Wound infection**	0	1	1	0
**Intra-abdominal abscess**	1	1	3	0
**Enteroatmospheric fistula**	0	0	3	0
**Anastomotic leakage**	0	0	2	3
**Stoma complication**	0	0	1	0

Leak occurred in five cases (25%) at a median of 5 days after primary surgery (range: 2–10 days) (Table 3). There were neither significant differences in VAC treatment duration nor in VAC pressure between patients with and without leakage. In one patient, the leakage was re-sutured with no subsequent leakage. The remaining four patients received a stoma. Four patients died within 90 days postoperatively (20%; 95% CI 6%-44%) (Table 3). None of these four patients experienced leakage. The causes of death were sepsis with multi-organ failure in one case, and three died from pulmonary complications. Thirteen of 16 patients (83%) who survived were discharged without a stoma. The rest had a permanent stoma.

**Table 3:** Postoperative complication rate

Complication [n (%)]	Total: 20 (100%)
Anastomotic lekage	5 (25%)
Mortality	4 (20%)
Fascial dehiscence	3 (15%)
Enteroathmospheric fistula	3 (15%)

In all patients, the fascia was closed after VAC termination. Three patients (15%) were re-operated for fascial dehiscence (Table 3). Three patients developed EAF (15%) (Table 3). One of the patients with EAF had an anastomotic leakage. There were no significant differences in negative pressure used for patients developing EAF compared to those without (with EAF median 75 (25;75), without EAF 50 (25;75). All EAFs were initially treated with resection and anastomosis, with a recurrence in all. No bleeding, stroke, acute myocardial infarction, pulmonary embolism, or deep vein thrombosis were observed in any patients.

## Discussion

The principle of suture or primary anastomosis and open abdomen with VAC treatment was feasible but had a high risk of anastomotic leakage and EAF formation after small intestinal perforation. The 90-day mortality rate of 20% was comparable with other reports on patients with secondary peritonitis treated with intestinal resection and proximal ostomy [1, 2].

After primary suturing or anastomosis, the leakage rate was high (25%). Another concern was related to the frequent development of EAF in 15% of cases, which was preceded by leakage in only one case. All cases with EAFs presented as an abdominal catastrophe, where conservative treatment was deemed too risky for the patient. They were treated with resection and anastomosis. Although all re-occurred this was with a less dramatic clinical presentation, where a symptomatic treatment with drainage, antibiotics and parenteral nutrition was possible and considered to be safe. A systematic review of different negative pressure techniques including commercial negative pressure kits, VAC-pack, negative pressure with sequential closure, Bogota Bag and Artificial Burr in the open abdomen reported a higher incidence of EAF formation in septic patients than non-septic patients (12.1% vs. 3.7%) [34]. A prospective analysis of the International Register of Open Abdomen (IROA) by Coccolini et al. included 649 patients treated with an open abdomen, and 51.2% of the patients were diagnosed with peritonitis [35]. EAF occurred in 12% of the peritonitis group but this incidence did not differ significantly from that in the control group without peritonitis. In another cohort study of 43 patients treated with VAC due to secondary peritonitis, an EAF rate of 37% was reported [31].

Pipariya et al. studied 50 patients with secondary peritonitis treated with primary repair/resection and compared this cohort with 50 patients who had undergone repair/resection with proximal stoma formation [36]. Non-traumatic perforation was the most common aetiology (61%) and most often confined to the terminal ileum (73%). The leak rate in the primary repair/resection group was 8%, and in the stoma group, approximately one-third developed stoma complications (prolapse, hernia, necrosis, and retraction). However, a critical study limitation was that patients in the stoma group had more severe peritonitis and were in poorer condition. A comparative study consisting of 30 primary repairs and 30 loop ileostomies after non-traumatic ileal perforation showed an increased rate of postoperative complication in the primary repair group; further 20% of the primary repairs had a leakage [37].

Our study limitations include its retrospective design and a relatively long inclusion period. Nevertheless, as the pathophysiology of peritonitis secondary to bowel perforation was similar, irrespective of the underlying cause, we found it relevant to perform this study even though the number of patients was relatively small. Furthermore, a limited number of surgeons performed all treatments at a single centre, making our approach to open abdomen and VAC uniform and comprehensive. In the 16 patients with an iatrogenic perforation, a missed enterostomy could be the reason. The charts were carefully reviewed, and all cases included a description with systematic inspection of the entire bowel for missed enterotomies at the end of the operation. The available patient physiologic parameters varied greatly within the pre- and postoperative period, and it was impossible to choose which parameter and at what time during the patient trajectory they should be registered. To our knowledge no single parameter has been shown to be clearly decisive for the right treatment in this group of patients. A scoring such as SFOFA could have been interesting to investigate, but this was only sporadically available. Finally, the number of patients is too small for a reliable statistical analysis.

## Conclusions

Primary anastomosis or suturing and open abdomen with VAC in patients with faecal peritonitis due to small intestinal perforation is associated with a high risk of anastomotic leakage and EAF formation. Therefore, the approach should only be considered for cases in which exteriorization of the intestine for stoma formation is difficult/impossible or may lead to short-bowel syndrome.

## Data Availability

The datasets used and/or analyzed during the current study available from the corresponding author on reasonable request.

## Abbreviations

VAC: Vacuum-assisted closure
EAF: Enteroatmospheric fistula
BMI: Body mass index
IROA: International Register of Open Abdomen
HP: Hartmann’s procedure
DCS: Damage control surgery
